# Silencing of *ABCC13* transporter in wheat reveals its involvement in grain development, phytic acid accumulation and lateral root formation

**DOI:** 10.1093/jxb/erw224

**Published:** 2016-06-23

**Authors:** Kaushal Kumar Bhati, Anshu Alok, Anil Kumar, Jagdeep Kaur, Siddharth Tiwari, Ajay Kumar Pandey

**Affiliations:** ^1^National Agri-Food Biotechnology Institute (Department of Biotechnology), C-127, Industrial Area, Phase VIII, S.A.S. Nagar, Mohali-160071, Punjab, India; ^2^Department of Biotechnology, Panjab University, Chandigarh, Punjab, India

**Keywords:** ABC-type transporters, cadmium stress, cadmium tolerance, lateral roots, phytic acid, *Triticum aestivum*.

## Abstract

Low phytic acid is a trait desired in cereal crops and can be achieved by manipulating the genes involved either in its biosynthesis or its transport in the vacuoles. Previously, we have demonstrated that the wheat TaABCC13 protein is a functional transporter, primarily involved in heavy metal tolerance, and a probable candidate gene to achieve low phytate wheat. In the current study, RNA silencing was used to knockdown the expression of *TaABCC13* in order to evaluate its functional importance in wheat. Transgenic plants with significantly reduced *TaABCC13* transcripts in either seeds or roots were selected for further studies. Homozygous RNAi lines K1B4 and K4G7 exhibited 34–22% reduction of the phytic acid content in the mature grains (T_4_ seeds). These transgenic lines were defective for spike development, as characterized by reduced grain filling and numbers of spikelets. The seeds of transgenic wheat had delayed germination, but the viability of the seedlings was unaffected. Interestingly, early emergence of lateral roots was observed in *TaABCC13*-silenced lines as compared to non-transgenic lines. In addition, these lines also had defects in metal uptake and development of lateral roots in the presence of cadmium stress. Our results suggest roles of TaABCC13 in lateral root initiation and enhanced sensitivity towards heavy metals. Taken together, these data demonstrate that wheat ABCC13 is functionally important for grain development and plays an important role during detoxification of heavy metals.

## Highlight

This study demonstrates the importance of the wheat ABCC transporter (TaABCC13) for achieving low phytic acid in grains, and substantiates its previously speculated role during heavy metal detoxification.

## Introduction

In plants, phytic acid (*myo*-inositol 1,2,3,4,5,6-hexakisphosphate; PA; IP_6_) is a major storage form of seed phosphate that chelates important micronutrients (Ca, Zn, Mg, Fe, Mn, etc.) and therefore reduces their bio-availability (Sandberg and Andersson, 1988; Raboy, 2001, 2009). One strategy for enhancing the bioavailability of micronutrients is by lowering the PA content in cereal grains (Raboy, 2009). PA biosynthesis takes place by sequential phosphorylation of the primary substrate, *myo*-inositol, in the cytosol (Greenwood and Bewley, 1984; Rasmussen *et al.*, 2010). PA is subsequently compartmentalized into vacuoles by multidrug resistance-associated transporters (Raboy, 2002; Regvar *et al.*, 2011). Previous studies suggest that lowering the seed PA level can be achieved either by targeting genes involved in its biosynthesis or its compartmentalization (Raboy, 2001; Rasmussen *et al.*, 2010; Sparvoli and Cominelli, 2015).

Multiple genes involved in different steps of PA biosynthesis have been targeted to achieve low phytate content in diverse cereals (Shi *et al.*, 2005; Kim and Tai, 2011; Ali *et al.*, 2013a,b). The maize *lpa1-1* and *lpa2-1* lines were the first mutants studied, and produced seeds that had 50% to 66% lower PA levels and high inorganic phosphate (Raboy *et al.*, 2000). The *lpa1-1* mutation was mapped to a locus encoding an ATP-binding cassette transporter C (*MRP4*) with a function in PA transportation and the *lpa2-1* mutation was mapped to a locus encoding an inositol phosphate kinase (*IPK1*) with a function in PA biosynthesis (Shi *et al.*, 2003, 2005). Mutants similar to maize *lpa1* with defects in MRP (ABCC type) transporter activity have also been recovered from Arabidopsis (*ABCC5*; Nagy *et al.*, 2009), barley (*ABCC1*; Dorsch *et al.*, 2003), soybean (*ABCC1*; Panzeri *et al.*, 2011), and rice (*MRP5*; Xu *et al.*, 2009). These observations have encouraged reverse genetic approaches in crop plants to further validate the role of ABCC transporters in lowering the PA content. For example, MRP4 in *Zea mays*, MRP1 in *Phaseolus vulgaris*, and MRP5 in *Oryza sativa* have been targeted to generate low phytate content in grain (Shi *et al.*, 2007; Li *et al.*, 2014). Among all *lpa* mutants, the *lpa1* mutations have been most predominantly examined, with breeding and genetic engineering efforts in multiple plants (Supplementary Table S1 at *JXB* online).

Initially, plant ABCC transporters were known for their roles in heavy metal sequestration and transport of glutathione conjugates, but subsequently they have been examined for their role in multiple physiological responses (Ishikawa *et al.*, 1997; Gaedeke *et al.*, 2001; Martinoia *et al.*, 2002). Correlations were then observed between ABCC transporter-mediated lowering of PA in cereal grains and their pleiotropic effects. For example, *MRP5* knockout in Arabidopsis plants not only results in a decrease of seed PA content, but also makes the plant more sensitive in its response towards hormones (Gaedeke *et al.*, 2001; Nagy *et al.*, 2009). Similarly, Arabidopsis ABCC1, ABCC2, and ABCC3 are well-known active glutathione conjugate transporters. In addition, ABCC1 is a potential folate and metal transporter whereas ABCC2 and ABCC3 are involved in chlorophyll catabolite transport (Lu *et al.*, 1997, 1998; Tommasini *et al.*, 1998; Frelet-Barrand *et al.*, 2008; Raichaudhuri *et al.*, 2009; Song *et al.*, 2010). Similarly, maize ABCC4/MRP4 performs multiple functions including flavonoid transportation and PA compartmentalization, and *ZmMRP4* mutants have altered root morphology (Goodman *et al.*, 2004; Shi *et al.*, 2007; Badone *et al.*, 2012).

Hexaploid wheat is one of the important food crops that has been improved for numerous traits through breeding efforts. The grains of wheat and other cereals store several essential inorganic nutrients and micronutrients compartmentalized in the aleurone (Regvar *et al.*, 2011; Singh *et al.*, 2013; Bhati *et al.*, 2014). Wheat mutants and landraces have been studied for seed PA, iron bioavailability, and agronomic performance (Guttieri *et al.*, 2004; Salunke *et al.*, 2012; Gupta *et al.*, 2015). Surprisingly, the pleiotropic effects observed in wheat due to lowering of PA are more drastic compared to other crop species with either *lpa1*/*2*/*3* mutations (Guttieri *et al.*, 2004; Gupta *et al.*, 2015).

Unlike in other crops, transgenic-based approaches have not been utilized and assessed to address the roles of candidate genes to develop low phytate in wheat. Wheat genes involved in PA biosynthesis or its possible transport,have been studied previously (Bhati *et al.*, 2014; Aggarwal *et al.*, 2015). It has also been demonstrated that expression of *TaABCC13* in a heterologous system conferred tolerance to heavy metals via the utilization of glutathione conjugates as substrates. In the present work, the functional role of ABCC13 in wheat was investigated by RNAi-mediated gene silencing by using a constitutive promoter. Silencing of *ABCC13* in transgenic wheat reduced PA in mature grains, and it caused developmental defects during grain filling. Furthermore, wheat transgenic roots showed sensitivity for the presence of cadmium (Cd) and phenotypic defects were observed. These results along with previous information for *TaABCC13* suggest its important role in wheat.

## Materials and methods

### RNAi vector preparation and *Agrobacterium*-mediated transformation

A monocot-specific RNAi vector pMCG161 (TAIR stock-CD3-459) was used for silencing of the wheat (*Triticum aestivum*) *ABCC13* gene (Zalewski *et al.*, 2010; Gasparis *et al.*, 2011). This vector confers selection of transgenic plants in the presence of the herbicide BASTA, and the RNAi cassette is expressed ubiquitously under the control of the CaMV 35S promoter. Primers were designed to amplify and clone a 394-bp conserved sequence from *TaABCC13* that was confirmed by sequencing (Chr 4B, 4D, and 5A; Supplementary Fig. S1). This nucleotide fragment was used to clone sense (*AscI*/*AvrII*) and anti-sense (*SpeI*/*SgfI*) strands following a one-step cloning method. The primer sequences used are listed in Supplementary Table S2. The cloning of sense and antisense sequences on either side of a rice waxy intron was subsequently confirmed using multiple restriction digestions and sequencing. Final constructs with proper insertion of sense and antisense sequences was transformed into *Agrobacterium tumefaciens* strain AGL-1. Positive colonies were screened and a single *Agrobacterium* clone harboring a confirmed *TaABCC13*:pMCG161 RNAi construct was used for wheat transformation.

Wheat transformation experiments were performed using *Agrobacterium*-mediated transformation with minor modifications (Przetakiewicz *et al.*, 2004), while the growth and culture media were used as per Patnaik and Khurana (2003) and Gasparis *et al.* (2011). Bread wheat (cv C306) was grown in an experimental field of the National Agri-food Biotechnology Institute, Mohali, India. Immature seeds were collected at 12–16 d after anthesis (DAA) and sterilized aseptically using HgCl_2_ (0.01% w/v). These sterilized seeds were used to rescue immature embryos using manual dissection under a laminar flow hood. About 700 dissected embryos, with their scutella facing up, were cultured on callus-induction medium consisting of MS medium with 2mg L^–1^ of 2,4-Dichlorophenoxyacetic acid (2,4-D). After 48h of incubation at 22 °C in the dark, explants were infected with an *Agrobacterium* suspension (OD_600_ = 0.4) carrying the *TaABCC13*:pMCG161 construct. The *Agrobacterium* suspension in liquid MS medium with Silwet (0.001% v/v) was poured drop by drop over the explants (Gasparis *et al.*, 2011). Following 3 d of co-cultivation, growing calli were rinsed 4–5 times with autoclaved MilliQ water to remove excess bacterial suspension, and sub-cultured for 4 weeks on callus-induction medium containing cefotaxime (200mg L^–1^). Healthy calli were transferred to selection and regeneration medium supplemented with 1mg L^–1^ Zeatin and 2mg L^–1^ of the herbicide BASTA (Gasparis *et al.*, 2011). At least four rounds of selection, each for 3–4 weeks, were performed for the regeneration of putative transformants. The plantlets were rooted in MS medium containing 2.5mg L^–1^ BASTA. Healthy and well-rooted plants were transferred to soil-pots for hardening. The shoots regenerated from independent calli were considered as independent integration events and assigned as T_0_ putative plants.

### Transgenic integration and genetic analysis

The plants those survived after hardening were selected for gene integration analysis through PCR. Total genomic DNA was isolated from fully expanded leaves of putative transgenic and non-transformed (control) plants using the DNeasy Plant Mini Kit–QIAGEN following manufacturer’s protocol. Two sets of specific primers were designed for amplification of the *bar* gene and the *OCS1* terminator region of RNAi cassette (Gasparis *et al.*, 2011) (Supplementary Table S2). Amplicon generated by PCR from T_0_ plants was purified and sequenced for confirmation. An individual tiller from each T_0_ plant was also screened by PCR to eliminate the possibility of chimera. Subsequently, seeds developed from the PCR-positive T_0_ plants were subjected to segregation analysis on 2mg L^–1^ BASTA that was optimized for cv C306 (control wheat) in hydroponic media. The T_1_ progenies derived from positive transformation events that fitted a 3:1 Mendelian segregation ratio based on BASTA selection and *bar* gene amplification were cultivated up to the T_2_ and T_3_ generations. Homozygous lines were selected using PCR in the T_2_ and T_3_ generations. These homozygous *TaABCC13* RNAi lines were maintained up to the T_4_ generation with consistent BASTA selection as well as by PCR-based screening. At the onset of flowering, the spikes were tagged on the day of anthesis for each line for subsequent observation on days post anthesis. The mature seeds from wild-type C306 and the RNAi lines were collected at approximately 56 d post anthesis and stored in cool and dry conditions.

### RNA extraction and confirmation of gene silencing using qRT-PCR

Tissue samples from selected homozygous T_4_ lines were snap-frozen in liquid nitrogen and stored at −80 °C until further analysis. Total RNA was extracted using the RNeasy Plant MiniKit (Qiagen, Valencia, CA, USA), as per the manufacturer’s instructions. Two micrograms of DNA-free total RNA were used for cDNA preparation. Reverse transcription reactions were performed using the Transcriptor First-Strand cDNA Synthesis Kit RT-PCR (Roche, USA) according to the manufacturer’s instructions. Primers used in the expression study are noted in Supplementary Table S2. Quantitative real-time PCR analysis was performed by following SYBR Green (QuantiFast^TM^ SYBR Green PCR kit, QIAGEN) chemistry using the ABI PRISM 7500 Fast Realtime Platform (Applied Biosystems). Target genes were amplified by a two-step PCR reaction with an initial denaturation at 95 °C for 5min followed by 40 cycles of 95 °C for 30s and annealing/extension at 60 °C for 30s. Relative quantification for fold-changes were calculated and Ct values were normalized against wheat *ARF* (ADP-ribosylation factor, AB050957.1) as defined previously by Bhati *et al.* (2014) and Alok *et al.* (2015) using the 2^–ΔΔCT^ method (Livak and Schmitteng, 2001). The specificity of the amplification was also assessed for each gene by dissociation curve analysis. A unique peak on the dissociation curve was confirmed for each gene.

### Phytic acid, free phosphate and total protein estimation

Total phytate in seeds was estimated by a colorimetric method using a K-PHYT kit (Megazyme, Inc, Bray, Ireland). Mature seeds from T_4_ homozygous lines were ground into a fine powder and extracted using 0.66N HCl for 6h with continuous stirring. Reactions were processed as described previously (Bhati *et al.*, 2014). Free phosphate in mature seeds of selected RNAi lines was measured by the ascorbate and ammonium molybedate method (Ames, 1966). Total protein was estimated by using the Bradford method (Bradford, 1976) with comparison against a BSA standard curve.

### Elemental analysis and cadmium uptake assays

Sterilized seeds of homozygous RNAi lines and non-transformed plants were germinated on Hoagland liquid media (Hoagland and Arnon, 1950) supplemented with 50 µM CdCl_2_. Sample collection, phenotypic observations, and Cd estimation were performed after 7 d. Root and leaf samples were heat-dried and microwave-digested with HNO_3_ (SuraPure^TM^, Merck). Total Cd uptake was measured by using Inductive Coupled Plasma-MS (ICP-MS) as described previously (Bhati *et al.*, 2014). In the figures the error bars indicate the standard deviation across two independent experiments.

## Results

### Homoeologous specific expression pattern of *TaABCC13*

*TaABCC13* was mapped to the long arm of the 4B, 4D, and 5A chromosomes with a similarity score of 99% and 97% (4B allele with respect to 4D and 5A). Fine mapping of these homoeologous sequences in the wheat genome assembly from the Ensembl plant server (http://plants.ensembl.org/Triticum_aestivum /Info/Annotation) helped in identification of their physical location on the respective chromosomes (Supplementary Table S3). The wheat ABCC13 transporter was conserved among orthologs from other cereals that share phylogenetic proximity (Supplementary Fig. S2). *TaABCC13* was differentially expressed during the developmental stages of wheat grains (Bhati *et al.*, 2014). To check the homoeolog-based expression of *TaABCC13*, gene-specific primers were used to determine the contribution from different wheat genomes. Expression of each of the *TaABCC13* homoeologs at two developmental stages during grain filling suggested preferentially high expression of transcripts arising from the B genome (Supplementary Fig. S3). The expression of all *TaABCC13* homoeologous transcripts was higher at 14 DAA than at 21 DAA. The transcript accumulation derived from the B genome was ~13-fold higher at 14 DAA compared to 21 DAA.

### Selection and screening of transgenic wheat lines

**Fig. 1.**
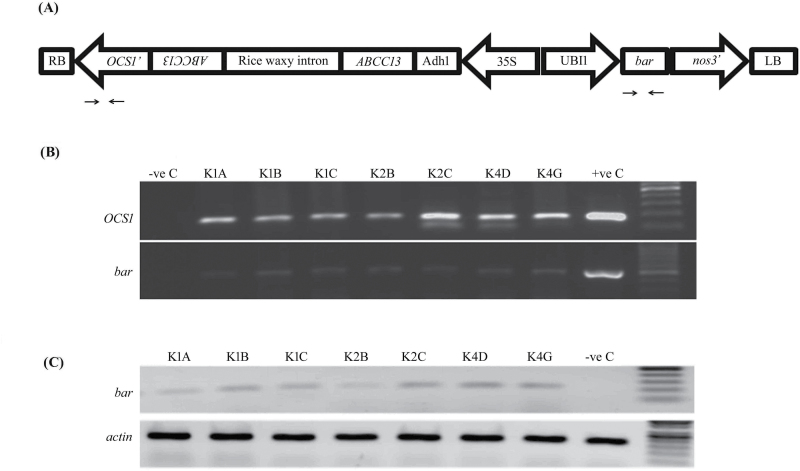
Designing the *TaABCC13* RNAi construct and selection of transgenic plants. (A) Schematic image of the pMCG161 vector backbone with inverted repeats of *TaABCC13* and the binding site for representative primers used for screening and selection of progeny positive for T-DNA integration. (B) Genomic DNA was isolated from the second leaf of T_1_ progeny. PCR reactions using primers targeting the *OCS* terminator (190bp) and the *bar* (480bp) gene were performed to confirm the presence of the transgene. The amplification products were visualized by using agarose gel electrophoresis. (C) cDNA was prepared from RNA isolated from the second leaf of PCR-positive plants from representative lines and *bar* gene-based primers were used for PCR amplification up to 30 cycles; the control reaction was run using the actin gene.

To gain insights into the function of *TaABCC13* in wheat, a binary vector pMCG161 with a constitutive promoter was designed to target a conserved region of the homoeologous gene sequences (Fig. 1A and Supplementary Fig. S1). The pMCG161 binary vector was previously shown to be an efficient vector for gene silencing in monocots such as barley, wheat, and maize (Zalewski *et al.*, 2010; Gasparis *et al.*, 2011). Nine independent putative transgenic events survived during multiple rounds of selection on BASTA (Supplementary Fig. S4), but only four of the putative transgenic plants survived the hardening procedure. These four putative transformants were subsequently confirmed for the presence of the transgene by amplifying and sequencing the *bar* and *OCS1* terminator sequences (Fig. 1B; Supplementary Fig. S5A, B). Additionally, mosaicism of transgenic integration in the wheat callus was also ruled out by screening each tiller by amplification and sequencing of *bar* (Supplementary Fig. S5C), and the flag leaves of T_0–1_ plants were found positive for *bar* transcript expression (Fig. 1C). The T_1_ progenies from the third event (K3) failed to survive due to reduced seed setting and subsequent failure of seed germination. Eventually, three independent transgenic events (K1, K2, and K4) showed healthy growth and seed germination for further analysis. The lines from these three independent events were propagated to the T_4_ generation, which was analysed in detail. Two transgenic plants from lines K1 (K1B4-2–5, K1A13-8-2), K2 (K2C4-6–8, K2C9-2–3), and K4 (K4G3-5-1, K4G7-10–3) selected randomly for further study.

### Silencing of *TaABCC13* in wheat RNAi lines

**Fig. 2.**
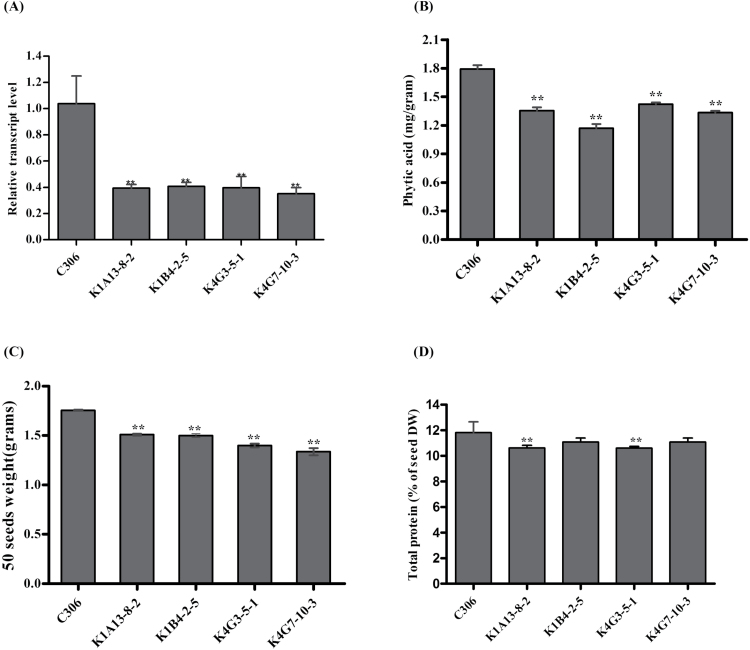
Silencing in seeds, phytic acid estimation, and seed quality of *TaABCC13*:RNAi lines. (A) Relative transcript levels of *TaABCC13* in different RNAi lines of wheat. Total RNA was isolated at 14 d after anthesis of the primary tiller of non-segregating T_4_ RNAi lines, cDNA was prepared from 2 µg of RNA (DNA free) and qRT-PCR analysis was performed using a SYBR Green-based assay. (B) Estimation of total phytic acid in mature wheat grains of transgenic lines. Seeds were collected from the primary tiller of each line. (C) Average seed weight of the *TaABCC13:*RNAi lines was determined by weighing 50 random seeds. (D) The total protein content of seeds from the *TaABCC13:*RNAi lines and non-transgenic parent was determined by using the Bradford method. Each bar indicates the mean of three biological replicates (three technical replicates). ** indicates significant differences at *P*<0.05 (*t*-test).

The selected T_3_ or T_4_ transgenic plants developed from three events (K1, K2, and K4) were subjected to qRT-PCR to assess the level of gene silencing in varying tissues. Transcript abundance of *TaABCC13* was quantified in T_4_ seeds at 14 DAA in the grains and flag leaf of the same tiller (Supplementary Fig. S6). The fold-decrease in the *TaABCC13* transcript for the T_4_ generation seed is presented in Fig. 2A and Supplementary Fig. S6. The silencing of *TaABCC13* resulted in 20–60% reduction in the transcript levels in the seed tissue, while 20–70% of silencing was observed in the flag leaf (Fig. 2A and Supplementary Fig. S6A, B). Maximum seed tissue silencing was observed for lines K4G7-10–3, K4G3-5-1, K1A13-8-2, and K1B4-2–5 in both T_3_ and T_4_ developing seeds. The representative silenced lines were further used for detailed characterization and phenotypic studies. As noted above, chromosome 4B is the major contributor of the *TaABCC13* transcripts (Supplementary Fig. S3), and we wanted to know how the silencing construct affected the expression of each of the homoeologs. To study the relative expression of the transcripts of *TaABCC13*, homoeolog-specific primers were employed and expression was compared to respective non-transgenic plants. The results confirmed that the construct was able to reduce the expression of the transcripts derived from the 4B, 4D, and 5A chromosomes by 40–72% (Supplementary Fig. S6C). In addition, to check whether other closely related *ABCC* genes (*TaABCC3* and *TaABCC4*) were affected by silencing of *TaABCC13*, expression analysis was performed for them. No significant change in the transcript of *TaABCC3* and *TaABCC4* was observed in the non-transgenic and transgenic lines (Supplementary Fig. S6D). This suggests that the silencing construct was specific for *TaABCC13* and probably does not affect the expression of the other studied *ABCC* genes.

### *TaABCC13* silencing reduces grain total PA content

To examine the effect of silencing of *TaABCC13* in seeds, PA content was measured in the mature grains. Significant differences in the accumulation of PA were observed among the transgenic lines that ranged from 22% to 34% reduction when compared to the non-transformed mature seeds. The maximal reduction in seed PA was observed in line K1B4-2–5 (~34%) followed by K4G7-10–3, which had a ~22% reduction in the PA level (Fig. 2B). No significant changes in total seed phosphorus (P) was observed for the *ABCC13*:RNAi lines as compared to C306 (Supplementary Fig. S7). However, the lowering of PA content was accompanied by a increase in free phosphate (Pi) for these transgenic lines (data not shown). Silencing of *TaABCC13* in the selected transgenic lines resulted in a slight reduction in seed weight (Fig. 2C). Although this slight reduction was observed consistently in other lines, it was most apparent in the K1B4-2–5 and K4G7-10–3 lines. The calculated protein content range for C306 was 11–12%. Although our transgenic lines varied in protein content from 10.3–11.2%, it was within the usual range expected for C306 (Fig. 2D).

Reduction in PA is often accompanied by remobilization or a change in the content of micronutrients (Ali *et al.*, 2013a). Transgenic wheat seeds showed increases in the accumulation of Ca, but no significant changes were observed for micronutrients such as Zn and Fe. The accumulation of Ca was ~1.7–1.8-fold greater than in the non-transformed control seeds (Table 1). Results of Perl’s staining showed a decreased density of iron at the crease region in the transgenic seed as compared to C306 (Supplementary Fig. S8). Enhanced colouration was also observed on the germinating coleoptile region, suggesting a possible early remobilization of iron in transgenic seeds.

**Table 1.** Estimation of metal content as analyzed by ICP-MS in the T_4_ grains of two wheat transgenic lines and the non-transgenic control (C306). The data are means ±SD with three biological replicates.

**Metal (µg g** ^**−1**^)	**C306**	**K1B4-2–5**	**K4G7-10–3**
Fe	47.2±0.15	55.8±0.21	45.9±0.05
Ca	75.2±1.09	80.2±0.35	82.38±0.30
Zn	22.6±0.20	25.22±0.56	23.2±0.06
Mn	48.06±0.77	45.42±0.50	48.3±3.00

### TaABCC13 affects grain filling, spike characteristics, and lateral root formation

**Fig. 3.**
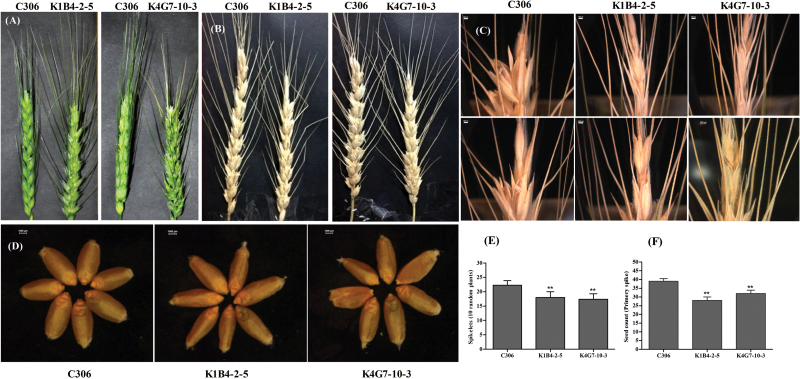
*TaABCC13* silencing affects grain filling and spike characteristics. (A) Representative images of the developing wheat caryopsis at the onset of flowering for C306 (control) and representative *TaABCC13:*RNAi lines. (B) Decreased length of spikes upon maturation for control (C306) and RNAi lines. (C) Detailed grain filling at the spike head on control (C306) and RNAi lines. (D) Representative images of seeds collected from C306 (control) and RNAi lines. (E) Total spikelet counts from RNAi lines as compared to C306. (F) Seed count from the primary spikes of the C306 and RNAi lines. Observations presented here were collected from T_4_ progenies that were consistent with the non-segregating lines of the T_3_ generation. The data in (E) and (F) are means ±SD. Ten spikes from each *TaABCC13:*RNAi line (only from the primary tiller) and the respective controls were considered for above observations. ** indicates significant differences at *P*<0.05 (*t*-test).

Previously, it was shown that wheat ABC transporters affect anther development and subsequently the process of grain maturation (Niu *et al.*, 2013; Walter *et al.*, 2015). Reports from multiple studies have also suggested that lowering PA generally results in altered grain morphology (Guttieri *et al.*, 2004; Li *et al.*, 2014). To assess the contribution of ABCC13 to grain development, developing spikes after heading were examined for phenotypic changes in C306 and transgenic plants. An altered spikelet arrangement was noticed in the developing spikes of transgenic lines. In these lines, the outer glumes are more exposed, thus causing spikes with an altered spikelet arrangement that is always accompanied by a reduction in total spikelet counts, when compared to the primary tiller of the control plants (Fig. 3A, B, E). These observations were consistently observed for both the silenced lines. The occurrence of head sterility was also observed in these RNAi lines in T_2_ to T_4_ progenies (Fig. 3C). Therefore, seed setting and grain filling were affected in transgenic lines, but the head development was normal at the pedicel region in both plants. Taking into account the pleiotropic effects of silencing *TaABCC13*, a reduction in the number of seeds recovered from each primary spike was generally observed (Fig. 3F). These results suggest the possibility that grain development directly involves ABCC13, or it could be affected by PA levels.

**Fig. 4.**
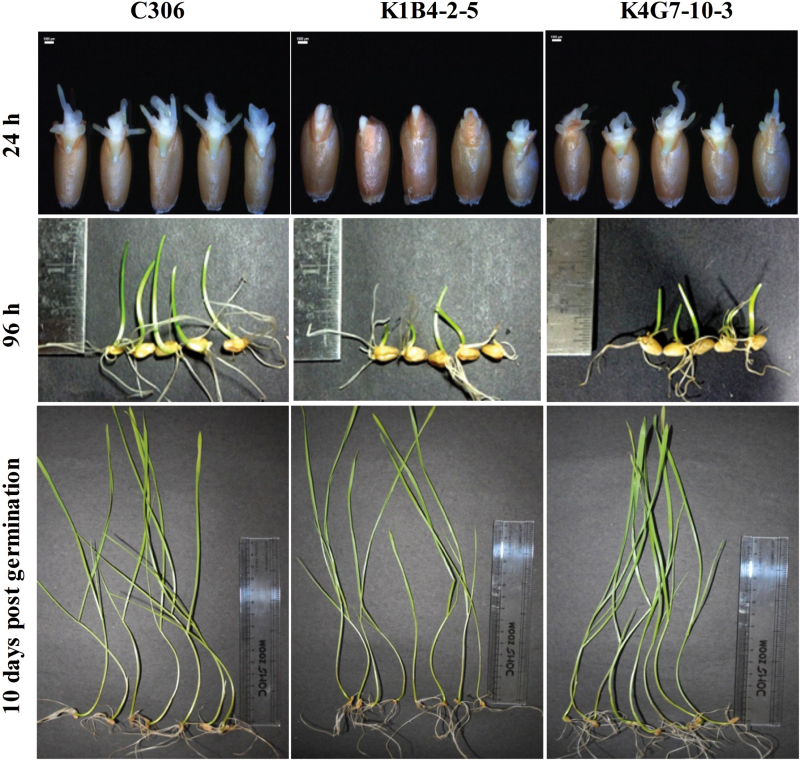
Germination of seeds from the *TaABCC13*:RNAi lines. Twenty seeds from mature plants (56 d after anthesis) from each transgenic line and control C306 plants were collected, sterilized, and soaked overnight in sterilized water. Seeds were then transferred to a growth chamber for germination. Representative images were photographed at 24h, 96h and at 10 d post germination.

A reduction in PA often correlates with the rate of germination for cereal grains. The germination rate of transgenic wheat (T_4_) with low PA levels was reduced by 5–7% when compared to the non-transgenic wheat seeds. Interestingly, a slow rate of germination was observed for low-PA seeds, which was apparent at the early times points (24 and 96h) after imbibition. In addition, we also observed that the germination rate was comparatively slow in transgenic compared to non-transgenic seeds during the early hours of germination. (Fig. 4). At a later stage of development (10 d), no significant difference in the height of germinated seedlings was noted between transgenic or non-transgenic lines (Fig. 4). These data confirm that silencing of *ABCC13* in wheat is related to lowering of total PA and to grain development.

**Fig. 5.**
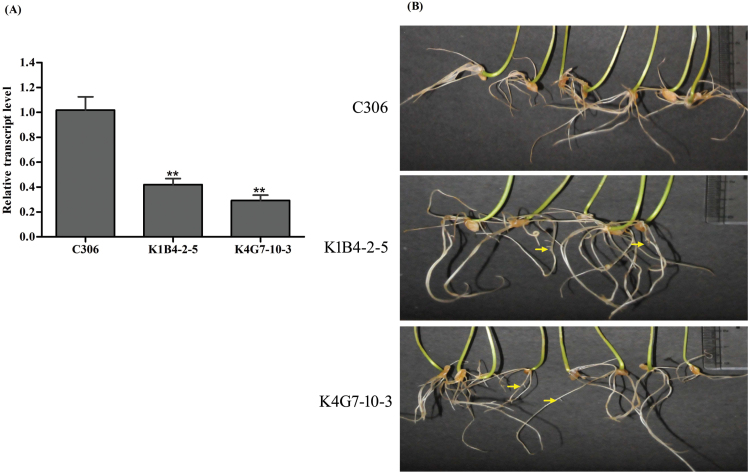
Emergence of lateral roots in *TaABCC13*:RNAi lines. (A) Relative transcript level of *TaABCC13* in different RNAi lines of T_4_ wheat seedlings. cDNA was prepared from 2 µg RNA (DNA free) and qRT-PCR analysis was performed using a SYBR Green-based assay. (B) Phenotypic analysis of the roots of C306 and transgenic RNAi lines. Ten seeds from *TaABCC13*:RNAi and C306 were germinated on half-strength Hoagland media in a hydroponic system and observations were recorded at 10 d after germination. Each bar indicates the mean of three biological replicates (10 technical replicates).

Plant ABCC transporters are also known for their role during root development under stress conditions (Gaedeke *et al.*, 2001). The K1B4-2–5 and K4G7-10–3 transgenic lines were selected to determine the effect of *TaABCC13* silencing on root development. In general, *TaABCC13* transcript was reduced by 30–65% in the roots of the transgenic lines (Fig. 5A), but there was no difference in the root length compared to the non-transgenic plants. Interestingly, early emergence of lateral roots was observed in transgenic seedlings in contrast to non-transgenic plants (Fig. 5B). The formation of lateral roots in the transgenic lines was higher as compared to the non-transgenic, suggesting that *TaABCC13* might be involved in controlling the emergence of lateral roots in wheat.

### Influence of cadmium toxicity on the wheat transgenic lines

**Fig. 6.**
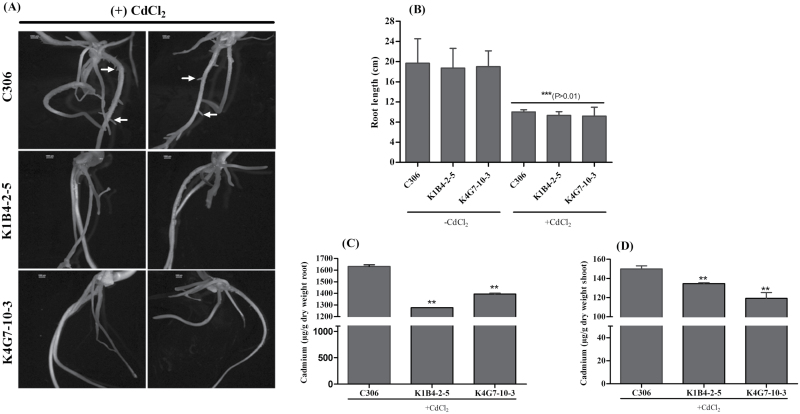
Differential root architecture and cadmium uptake assay in RNAi seedlings. (A) Phenotypic differences were observed on the roots of C306 and *TaABCC13*:RNAi lines. Ten seedlings from transgenic and control lines were exposed to 50 µM CdCl_2_ for 7 d. After sprouting, seedlings were transferred to a hydroponic system for 7 d of heavy metal stress treatment. Roots were observed under a light microscope. Representative images are presented with two replicate plantlets after 7 d exposure to Hoagland media supplemented with cadmium. (B) Primary root length was measured at 7 d after C306 and RNAi plantlets were exposed to heavy metal stress. Seedlings without heavy metal exposure were used as the experimental control. (C, D) Cadmium uptake in roots (C) and shoots (D) at 7 d after exposure to CdCl_2_. Samples were washed using ICP-MS grade water after collection from the hydroponic system, heat-dried and digested in HNO_3_ using high-intensity microwaves. PPM outputs from the ICP-MS run were normalized with ICP-MS values of the controls with cadmium stress. The data in (C) and (D) are means ±SD. ** indicates significant differences at *P*<0.05 (*t*-test).

Heterologous expression of *TaABCC13* in yeast has suggested a role in Cd tolerance that could utilize glutathione conjugates as substrates (Bhati *et al.*, 2014, 2015). In order to further characterize the role of *TaABCC13* in heavy metal detoxification, wheat RNAi transgenic seedlings were exposed to Cd. As expected, the emergence of multiple lateral roots was observed in control plants treated with Cd. Transgenic lines showed phenotypic sensitivity when exposed to Cd compared to non-transgenic (Fig. 6A). Interestingly, the *TaABCC13*:RNAi lines did not develop lateral roots when exposed to Cd (Fig. 6A). This phenotype was noted in two different transgenic lines (K1B4-2–5 and K4G7-10–3). In general, a decrease in the root length was observed in Cd-exposed plants when compared to untreated seedlings (Fig. 6B). The shoot biomass was significantly higher in C306 plants as compared to *TaABCC13*:RNAi seedlings exposed to Cd (Supplementary Fig. S9). The *TaABCC13* silencing and altered root phenotype under Cd stress resulted into differences in the root and shoot uptake of Cd (Fig. 6C, D). This *in planta* evidence shows that Cd uptake is reduced and sensitivity to Cd is decreased in *TaABCC13:*RNAi lines, thus signifying the importance of ABC transporters for Cd detoxification.

## Discussion

The present study provides insights into multiple physiological roles of ABCC13 in wheat. Previous studies have proposed high expression levels of *TaABCC13* in immature seeds and roots, thus advocating *TaABCC13* as a potential candidate gene regulating seed and root phenotypes in wheat (Bhati *et al.*, 2015). The constitutive expression of the RNAi cassette targeting *TaABCC13* enabled the functional validation of this gene not only in seeds but also in the root tissue. *TaABCC13* is evolutionarily conserved with orthologs of other candidate genes from maize (*ZmMRP4*) and Arabidopsis (*AtMRP5*) that were demonstrated to have multiple functions in earlier studies (Gaedeke *et al.*, 2001; Badone *et al.*, 2012). In the present study, we observed that *ABCC13*-silenced wheat transgenic lines had reduced PA and altered heavy metal detoxification.

### ABCC13 as a candidate to achieve low phytic acid in wheat

Multiple approaches have been considered to generate a low-phytate trait in crop plants such as soybean, rice, and maize (Shi *et al.*, 2007; Kim and Tai, 2011; Ali *et al.*, 2013a,b; Li *et al.*, 2014). However, such studies have not previously been undertaken for important crops such as wheat where most of the PA–along with micronutrients–accumulates in the aleurone layer (Bohn *et al.*, 2008; Bhati *et al.*, 2014). Utilizing a transgenic approach to modify the PA in wheat is important since genetic variation for this trait in this species is very limited. Although the transcript arising from chromosome 4B was highly expressed, to evaluate the functionality of *TaABCC13* a conserved region of the three homoeologous genes was targeted (Supplementary Figs S1 and S6). In general, we observed that this construct was able to reduce relative expression levels of all the transcripts of *TaABCC13* irrespective of the origin.

Recently, wheat genes involved in biosynthesis of PA have been reported, and these could be potential candidates for developing low-PA traits (Bhati *et al.*, 2014). ABCC transporters are involved in the transport or compartmentalization of the PA after its synthesis in grains. Loss-of-function mutations for ABCC transporters have been characterized with varying levels of reduction in seed phytate (Raboy *et al.*, 2000; Pilu *et al.*, 2003; Raboy, 2009; Panzeri *et al.*, 2011). Moreover, *ABCC13* also shares close homology with *ZmMRP4* and *AtMRP5* (90.5 and 73.5%, respectively), with a similar exon–intron arrangement (Bhati *et al.*, 2014). These studies strongly support this ABCC transporter as a strong candidate for the development of a *lpa* phenotype in agronomically important crops.

Wheat RNAi lines targeting this *ABCC* transporter gene (K1B4-2–5 and K4G7-10–3) showed 34–22% reduction in the PA level with a concomitant increase in calcium. Depending on the crop and the candidate genes targeted, lowering of PA is often accompanied by an increase in micronutrients, as demontsrated in other studies (Shi *et al.*, 2007; Ali *et al.*, 2013a). In our transgenic seeds, although no significant increase in the iron content was observed, our preliminary screening suggested its faster remobilization in other tissues. This phenomenon was consistently observed in most of our RNAi seeds, reinforcing the correlation between the rate of iron remobilization and lowering of PA. Studies utilizing spectroscopy-based methods could be further used to validate these redistribution patterns.

The only previous report for reduced PA in wheat was a line with mutations in two independent *lpa1*-related loci that caused a 37% reduction (Guttieri *et al.*, 2004). Similar, the *lpa-1* mutation in barley and maize showed reduction of 50–95% and 50–60%, respectively (Raboy *et al.*, 2000; Dorsch *et al.*, 2003). Reduction of PA due to a defective PA transport mechanism causes significant pleiotropic effects that results in unacceptable agronomic field performance in wheat, maize, and rice (Guttieri *et al.*, 2004; Badone *et al.*, 2012; Li *et al.*, 2014). Some of these negative impacts were also observed in the current study in *TaABCC13*:RNAi lines, including reduced seed weight, slightly delayed germination, and slow coleoptile growth. However, these defects were not as drastic as reported in the first wheat *lpa* mutant and in the low field performance by the *lpa1-7* mutation in *ZmMRP4* (Guttieri *et al.*, 2004; Badone *et al.*, 2012). Similar agronomical impacts of reduced PA have been observed in other crop plants silenced for genes involved in either the early or late phase of PA biosynthesis (Raboy, 2009; Ali *et al.*, 2013a,b; Sparvoli and Cominelli, 2015). Thus these impacts may vary from crop to crop, and with the choice of the candidate gene. Phenotypic effects on the *lpa* crops could be reduced by utilizing tissue-specific targeting of the PA biosynthetic pathway (Shi *et al.*, 2007; Ali *et al.*, 2013a). Such strategies could also be designed to target *ABCC13* in the aleurone to develop an *lpa* trait in wheat, because the aleurone is the tissue where the *TaABCC13* transcript is abundant (Bhati *et al.*, 2014).

### Role of TaABCC13 in grain development and physiological characteristics

The phenomenon of multi-functionality is common among plant ABCC transporters (Gaedeke *et al.*, 2001; Nagy *et al.*, 2009; Walter *et al.*, 2015). Functional studies of the wheat ABCC transporter have established its roles in responses to fungal pathogens, and in grain formation and maturation (Walter *et al.*, 2015). Subsequently, multiple wheat ABCC transporters were reported that are highly expressed in developing grains (Bhati *et al.*, 2015). These reports have emphasized the need to expand our knowledge of the role of ABCC transporters in wheat. Our data confirm the importance of *TaABCC13* and generally support the emerging roles of ABCC transporters in grain development and maturation. The early development of wheat spikes is regulated by abscisic acid (ABA) and gibberellic acid (GA) responses (Thiel *et al.*, 2008; Pearce *et al.*, 2013). Previously we have reported that the exogenous application of GA significantly stimulated *TaABCC6*, *TaABCC8*, and *TaABCC13* transcript levels in wheat seeds (Bhati *et al.*, 2015). Plant ABCC transporters are known to have a role in the transport of derivatives in hormone biosynthesis to further facilitate localized hormonal response (Gaedeke *et al.*, 2001; Ko *et al.*, 2014; Borghi *et al.*, 2015). Inositol phosphate biosynthesis pathways control signaling responses in eukaryotic cells (Gillaspy, 2013). In plants PA also acts as a signaling molecule or a co-factor that controls the physiological response to hormones or oxidative stress (Lemtiri-Chlieh *et al.*, 2003; Tan *et al.*, 2007; Doria *et al.*, 2009; Sparvoli and Cominelli, 2015). The biosynthesis of wheat PA starts at the early stages of seed development. Therefore, one could speculate that the phenotypes observed during wheat spike development are possibly effects of reduced PA signaling and/or perturbation in the ABCC13-dependent transport of hormonal derivatives.

Plant roots may develop different phenotypes and anatomical features when exposed to metal stress. Higher plants commonly develop lateral roots in an attempt to block the radial transport of heavy metals (Hu *et al.*, 2013; Sofo *et al.*, 2013). In our experiments, non-transgenic wheat lines also developed lateral roots under Cd stress, reinforcing the conserved mechanism of sensitivity in plants. Plant ABCC transporters may have direct or indirect roles in regulation of plant development. Interestingly, the untreated transgenic lines silenced for *TaABCC13* showed an early emergence of lateral roots, but they were inhibited in the presence of Cd (Figs 5 and 6). Low PA mutants of maize (*zmmrp4*) and Arabidopsis (*atmrp5*) also have altered root phenotypes that include the early emergence of lateral roots (Gaedeke *et al.*, 2001; Nagy *et al.*, 2009; Badone *et al.*, 2012). The early emergence of lateral roots on *TaABCC13*:RNAi lines suggests a conserved role for *TaABCC13* in wheat root development. These observations coupled with previous evidence suggest a correlation for the possible homeostatic role of TaABCC13 in lateral root formation and metal stress.

It may be speculated that altered root development might occur as a result of changes in auxin flux (Gaedeke *et al.*, 2001). If this is the case, then we could propose that TaABCC13 may function in the transport of auxin or a derivative; however, our experiments do not directly test this hypothesis. In our study, 10-d-old C306 seedlings formed lateral roots when exposed to Cd, whereas *TaABCC13*-silenced plants were not able to develop lateral roots. This surprising reversal of lateral root development on *TaABCC13*:RNAi seedlings under Cd stress suggests that there is a complex interaction between the processes regulating root development and responses to Cd stress. In plants, the response activated by metals is mediated through the biosynthesis of signalling molecules such as phytohormones. Multiple reports have suggested that Cd interferes with the maintenance of auxin homeostasis (Xu *et al.*, 2010; Elobeid *et al.*, 2012; Hu *et al.*, 2013). Previous reports have also suggested that Cd alters the expression of multiple genes responsible for auxin biosynthesis and distribution, which leads to increased lateral root density (Hu *et al.*, 2013, Yuan and Huang, 2015). Our data not only validate the role of TaABCC13 during lateral root formation, but also link with the possibility for metal–auxin homeostasis.

The current study provides *in planta* evidence for the previously speculated role that TaABCC13 has in yeast. Taken together, our data reinforce the importance of targeting ABCC transporters to achieve low PA, and we further demonstrate their roles in heavy metal detoxification. This research lays the groundwork for further examination for the functions of ABCC transporters in wheat, especially those that are highly expressed during grain development.

## Supplementary data

Supplementary data are available at *JXB* online.

Table S1. List of ABCC transporters reported from different plant systems together with the approaches used to develop the low phytic acid trait.

Table S2. List of primers used in the present study.

Table S3. Physical coordinates of *TaABCC13* on the wheat genome on the Ensembl Plants genome database.

Figure S1. Multi-alignment of nucleotide sequence of *TaABCC13* transcripts used to design RNAi targets.

Figure S2. TaABCC13 is evolutionary close to *lpa1* orthologs from other cereals.

Figure S3. Differential expression analysis of three homoeologous of *TaABCC13* at developmental stages of the seed.

Figure S4. Representative images of regenerated transgenic seedling selected over herbicide BASTA with respect to the control.

Figure S5. Confirmation of RNAi integration in T_0_ transgenic plants.

Figure S6. Primary screening for relative transcript level for *TaABCC13* in multiple RNAi lines.

Figure S7. Estimation of total phosphorus in flour of *TaABCC13*:RNAi seeds and cv C306.

Figure S8. Perl’s staining for iron localization in cv C306 and transgenic seeds (low phytic acid content).

Figure S9. Effect of Cd stress on seedling biomass.
